# Investigation of Glucose Metabolism by Continuous Glucose Monitoring and Validation of Dipeptidyl Peptidase 4 Inhibitor Use in Patients with Myotonic Dystrophy Type 1

**DOI:** 10.3390/jcm13175252

**Published:** 2024-09-05

**Authors:** Hiroto Takada, Tsuyoshi Matsumura, Haruna Shimamura, Misa Matsui, Seiko Kon, Aono Fukumoto, Tomoya Kubota, Kosuke Yoshida, Hiromi Iwahashi, Masanori P. Takahashi

**Affiliations:** 1 Department of Neurology, NHO Aomori National Hospital, Aomori 038-1331, Aomori, Japan; takada.hiroto.yw@mail.hosp.go.jp (H.T.); kon.seiko.zc@mail.hosp.go.jp (S.K.); 2 Department of Neurology, NHO Osaka Toneyama Medical Center, Toyonaka 560-8552, Osaka, Japan; matsumura.tsuyoshi.kq@mail.hosp.go.jp (T.M.); matsui.misa.dn@mail.hosp.go.jp (M.M.); 3 Clinical Neurophysiology, Department of Clinical Laboratory and Biomedical Sciences, Osaka University Graduate School of Medicine, Suita 565-0871, Osaka, Japankubota.tomoya.sahs.med@osaka-u.ac.jp (T.K.); 4 Department of Neurology, NHO Asahikawa Medical Center, Asahikawa 070-8644, Hokkaido, Japan; koyoshida-hok@umin.ac.jp; 5 Department of Internal Medicine, Toyonaka Municipal Hospital, Toyonaka 560-8565, Osaka, Japan

**Keywords:** incretin, teneligliptin, hypoglycemia, myotonic dystrophy, diabetes

## Abstract

**Objectives:** We characterized blood glucose fluctuations in patients with myotonic dystrophy type 1 (DM1). After confirming the incretin secretion capacity of patients with DM1, we intended to clarify whether dipeptidyl peptidase 4 (DPP-4) inhibitor administration was appropriate in cases of DM1 with diabetes mellitus. **Methods:** A 48 h continuous glucose monitoring (CGM) was performed in 29 Japanese patients with DM1. An oral glucose tolerance test (OGTT) was performed in patients with DM1 and five disease controls, and levels of blood glucose, insulin, and incretin (glucagon-like peptide-1 and gastric inhibitory polypeptide) were measured. DPP-4 inhibitors were administered to patients with diabetes mellitus complicated by DM1, and the CGM results were compared. **Results:** The CGM showed distinct patterns of blood glucose variability among patients classified by an OGTT pattern with significant differences in glucose parameters such as time above 140 mg/dL and mean amplitude of glycemic excursions between the groups. High sensor glucose values were observed in a certain number of patients who were classified as having normal or impaired glucose tolerance by the OGTT. The CGM confirmed the presence of low glucose levels in several patients. Incretin secretion, the target of DPP-4 inhibitors, was preserved in patients with DM1. DPP-4 inhibitor treatment resulted in lower glucose levels and improved insulin secretion in some patients. **Conclusions:** This is the first CGM study for DM1 patients. The CGM identified potential early abnormalities in glucose metabolism in DM1. In the future, it will be crucial to explore effective methods for harnessing CGM and assessing it quantitatively in DM1.

## 1. Introduction

Myotonic dystrophy type 1 (DM1) is the most common type of muscular dystrophy in adults [1]. In addition to muscle wasting and myotonia, DM1 is associated with systemic features, including cataracts, cognitive dysfunction, cardiac conduction defects, and glucose intolerance [1]. DM1 is caused by an abnormal CTG repeat expansion in the *DMPK* gene. Pre-mRNAs containing expanded repeats aggregate in the nucleus and cause quantitative and qualitative changes in splicing regulators, leading to defects in various genes [2,3]. An increase in the levels of the mis-spliced isoform of the insulin receptor with low signaling (IR-A) is reported [3].

Insulin resistance is commonly observed in patients with DM1 [4]. Insulin resistance is thought to be partially due to abnormal IR-A expression. Therefore, impaired glucose tolerance in DM1 may show different clinical features from those of type 2 diabetes mellitus in the general population [5]. We have shown that diabetes mellitus can develop in patients with DM1 even with fasting blood glucose levels lower than those in patients with type 2 diabetes [6], and that blood glucose rises in the afternoon and evening [7]. Although minor, a significant proportion of patients require pharmacological intervention (more than 10% of the patients in the Japanese national registry) [8,9,10]. To provide the appropriate treatment for DM1 glucose intolerance, it is necessary to understand blood glucose variability in patients with DM1.

Glycated hemoglobin (HbA1c), which indicates the average blood glucose level over the past 2–3 months, is used as the gold standard for blood glucose control in diabetes care. It is impossible to identify diurnal blood glucose variations and hypoglycemia using HbA1c levels [11,12]. Self-monitoring of blood glucose (SMBG) is employed to monitor blood glucose fluctuations. Although it is possible to detect hyperglycemia, it is challenging to monitor hypoglycemia [13]. In contrast, continuous glucose monitoring (CGM), which has been widely applied in clinical settings in recent years, is helpful in monitoring glucose trends, including hypoglycemia [14,15]. CGM measures glucose in the interstitial fluid every 1–5 min. Glucose levels in the interstitial fluid correlate well with those in plasma glucose, despite a time lag of 10–15 min between the two. Herein, we perform CGM for patients with DM1 to capture glucose level variations.

The role of diet and exercise therapy in the treatment of diabetes associated with DM1 is limited, owing to manifestations such as muscle weakness and dysphagia [8]. Therefore, drug therapy is often necessary; however, a standard medication therapy has not been established. Thiazolidinediones such as pioglitazone and biguanides such as metformin are theoretically suitable for treating abnormal glucose metabolism in DM1 [9,16,17]. Metformin has been recently reported to improve splicing abnormalities in the skeletal muscle and mobility of patients with DM1 [17,18]. A relatively new medication, a DPP-4 inhibitor that promotes incretin action, is widely being used in Japan for patients with DM1 [10]; however, its effects have not yet been comprehensively investigated. Additionally, although DPP-4 inhibitors are less likely to cause hypoglycemia [19], the risk of hypoglycemia with DPP-4 inhibitor use in patients with DM1, where hypoglycemia is observed even in untreated cases, has not yet been clarified. Therefore, another objective of this study was to confirm whether the incretin secretion capacity, which is essential for the action of DPP4-inhibitors, is preserved in patients with DM1, and to evaluate the effect of DPP-4 inhibitors in patients with DM1 complicated by diabetes using CGM.

## 2. Methods

### 2.1. Subjects

The study was approved by the ethics committees of all institutions where the study was conducted. Patients aged 18 years or older at NHO Aomori Hospital, Osaka Toneyama Medical Center, and Asahikawa Medical Center were included in the study after written informed consent was obtained.

Patients with DM1 after genetic confirmation and disease controls (amyotrophic lateral sclerosis (ALS) or other neuromuscular diseases) were enrolled. Patients with a prior use of insulin, DPP-4 inhibitors, or glucagon-like peptide-1 (GLP-1) receptor agonists within the past three months or with a history of cardiovascular events were excluded. Patients with hepatic dysfunction (AST > 100 IU/L), renal dysfunction (estimated glomerular filtration rate < 45 mL/min/1.73 m^2^), or heart failure (NYHA classification III or higher) were also excluded.

This study was conducted on an inpatient basis to control the calorie intake of patients. Each hospital provided an inpatient diet, with calories ranging from 20 to 30 Cal/kg × IBW (ideal body weight, height(m)^2^ × 22). Variation in the percentages of carbohydrates, proteins, and fats between meals (breakfast, lunch, and supper) was kept between 5 and 10%. Food intake, time of meal taken, and total daily caloric intake were recorded. The patients were instructed to avoid snacking and to maintain the same physical activity level as usual.

### 2.2. Background Information

Patients’ heights and weights were also measured. Motor function was assessed using the modified Rankin Scale. The CTG repeat number of the DMPK gene, measured by Southern blotting, was obtained from medical records. Cystatin C and HbA1c levels were measured during fasting. Triglyceride, HDL-cholesterol, LDL-cholesterol, and free fatty acid levels were measured before breakfast and 2 h after breakfast.

### 2.3. Continuous Glucose Level Measurements by CGM

CGM was performed on patients with DM1 only. On day 1, a CGM device (iPro2, Medtronic, Tokyo, Japan) was placed on each patient’s abdomen at least four days after admission, and glucose sensor levels were continuously measured for 72 h. On the fourth day, the CGM device was removed after a 75 g oral glucose tolerance test (OGTT) was performed on patients after skipping breakfast. For calibration and confirmation of the CGM data, blood glucose levels were measured periodically using an SMBG device or venous blood sampling.

The mean glucose sensor value, mean amplitude of glucose sensor value (MAGE), percentage time of low glucose sensor value, percentage time of high glucose sensor value, and other indices were calculated using the CGM data recorded over the first 48 h of measurement. Because non-diabetic subjects were included, glucose sensor levels of 70–140 mg/dL were used as the reference range, following the criteria for blood glucose levels at any time. We also employed glucose sensor levels of 60 mg/dL in addition to 70 mg/dL to assess the low glucose level.

### 2.4. OGTT and Incretin Secretion Analysis

An OGTT was performed on the morning of the fourth day of CGM. Blood samples were drawn before loading and 30, 60, 90, and 120 min after loading. Blood glucose, immunoreactive insulin (IRI), and glucagon levels were measured at all five sampling points, whereas GLP-1 and gastric inhibitory polypeptide (GIP) levels were measured before, 30 min after, and 120 min after loading. Samples for GLP-1 and GIP measurements were collected in blood tubes containing protein stabilizers (Becton Dickinson Japan, Tokyo, Japan). GLP-1 and GIP levels were measured by ELISA using a Glucagon-Like Peptide-1 (Active) ELISA Kit 96-Well Plate (Merck Millipore, Billerica, MA, USA) and a Human GIP Active Form Assay Kit (Immuno-Biological Laboratories, Fujioka, Japan), respectively.

Based on the results of the OGTT, the patients were classified into three groups: normal (NGT), borderline (IGT), and diabetic (DM). The borderline type (IGT) was defined based on an FBS of 110–125 mg/dL or a blood glucose level of 140–199 mg/dL, 120 min after the OGTT. The diabetic type (DM) was defined based on an FBS of ≥126 mg/dL, or a blood glucose level of ≥200 mg/dL at 120 min after the OGTT. The insulinogenic index (II) was determined from the OGTT results as changes in blood insulin (at 30 min–at 0 min)/changes in blood glucose (at 30 min–0 min).

### 2.5. Investigation of the Effects of DPP-4 Inhibitor

The efficacy of a DPP-4 inhibitor, teneligliptin, was evaluated in DM1 patients with diabetes who met the following two conditions: (1) fasting blood glucose > 130 mg/dL or HbA1c > 7.0% to < 10.0%, and (2) fasting blood C-peptide > 1.0 ng/mL.

Two days after removing the CGM device (day six after the initial CGM device placement), teneligliptin, 20 mg once in the morning, was introduced, and the CGM was again placed for 72 h, starting at noon the following day. An OGTT was performed on the same schedule as the initial CGM, but only blood glucose and IRI were measured.

### 2.6. Statistical Analysis

Statistical analysis was performed using JMP software version 17 (JMP Statistical Discovery, Cary, NC, USA). The results are expressed as the mean ± standard deviation, unless otherwise noted. The differences between groups were assessed by the Wilcoxon signed-rank sum test. To assess the correlation, Spearman’s correlation coefficient was calculated. *p*-values less than 0.05 were considered statistically significant.

## 3. Results

Twenty-nine patients with DM and five disease controls (four with amyotrophic lateral sclerosis and one with multifocal motor neuropathy) were enrolled in this study. Table 1 shows the background information and glucose and lipid metabolism test results for all DM1 patients, the groups based on the OGTT results, and the control patients. Of the five patients in the disease control group, one had NGT, one had IGT, and three had DM.

### 3.1. Sensor Glucose Profiles Recorded by CGM and Its Parameters in DM1 Patients

**Figure 1:**
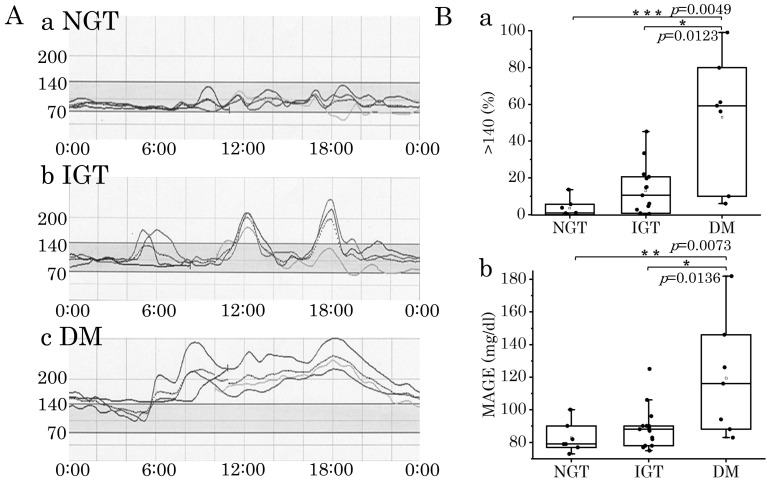
CGM profiles and parameters in DM1 patients. (**A**). Representative examples of sensor glucose profiles for patients classified as NGT (**a**), IGT (**b**), or DM (**c**). (**B**). CGM parameters, %time of glucose sensor values > 140 mg/dL (**a**) and MAGE (**b**) were compared for the three groups classified by OGTT. *, **, and *** indicate *p* < 0.05, *p* < 0.01 and *p* < 0.005 by Wilcoxon test, respectively.

Representative examples of sensor glucose profiles recorded by CGM for patients classified as NGT, IGT, or DM are shown. A patient with NGT (HbA1c 5.6) showed glucose sensor levels fluctuating almost exclusively within the normal range (70–140 mg/dL) during the day (Figure 1A(a)). A patient with IGT pattern showed glucose levels gradually increasing from morning to evening and deviating from the normal range (70–140 mg/dL) after a meal (Figure 1A(b)). Of note, although the HbA1c value was low at 5.1 in this patient, the OGTT showed IGT, and the CGM showed a DM pattern. A CGM profile in a patient with a DM pattern with an OGTT and an HbA1c of 7.0 showed that glucose levels rise after breakfast, do not fall, and remain elevated, with deviation from the normal range (70–140 mg/dL) (Figure 1A(c)).

We compared CGM parameters, including the %time of glucose sensor values > 140 mg/dL and the MAGE between the three OGTT groups. DM1 patients with DM had significantly higher values than those with NGT and IGT (Wilcoxon test). However, there was no significant difference between the NGT and IGT groups (Figure 1B(a,b)). Even within the NGT group, there was a patient where the %time of glucose sensor values > 140 mg/dL reached 14%, and there was considerable overlap between the IGT and NGT groups.

In the patient of Figure 1A(a), glucose sensor levels below 70 mg/dL were observed after dinner (roughly 18:00–20:00). Similarly, CGM data confirmed the presence of low glucose values in several patients (Table 2). Among the patients with NGT, we identified low glucose levels in six of seven cases. Of these, two showed glucose sensor values below 60 mg/dL. In patients with IGT, low glucose levels were confirmed in four of the fifteen patients, among which two showed sensor values below 60 mg/dL.

### 3.2. Correlation of CGM Parameters

**Figure 2:**
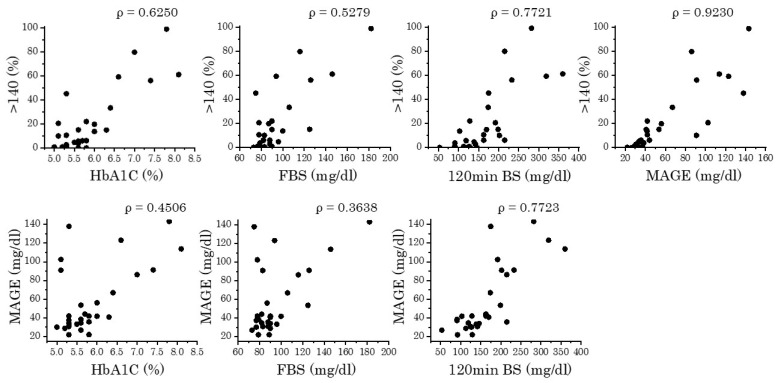
Correlation of CGM parameters. The correlation of CGM parameters, %time of glucose sensor values > 140 mg/dL and MAGE, and HbA1c, FBS and blood glucose level at 120 min in the OGTT are shown. *ρ*: Spearman’s rank correlation coefficient.

The CGM parameters, the %time of glucose sensor values > 140 mg/dL, and the MAGE were well correlated (Figure 2 top right). When we examined the correlation between these two CGM parameters and the HbA1c, FBS, and blood glucose level at 120 min in the OGTT (120 min BS), the highest correlation was found with 120 min BS (Figure 2).

### 3.3. Incretin Secretion in DM1 Patients

**Figure 3:**
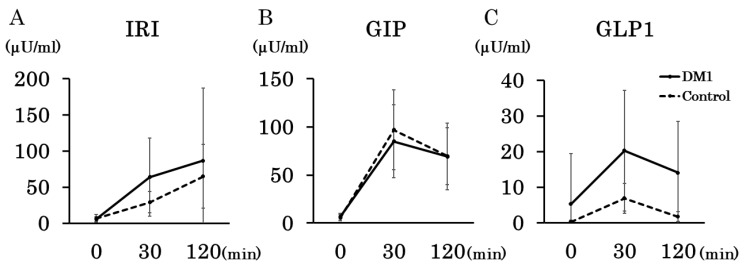
Comparison of insulin and incretin secretory capacity between patients with DM1 and disease controls. IRI (**A**), GIP (**B**), and GLP-1 (**C**) levels were compared between DM1 patients and disease-control with diabetics during the OGTT study. The DM1 group showed similar or higher values than the controls. In particular, there was a difference between patients with DM1 and controls in the 30 and 120 min values of GLP1.

Regarding the secretion of incretin, GLP1, and GIP, the twenty-eight patients with DM1, excluding one missing case, were compared with five diabetic control patients with neuromuscular diseases. The DM1 patients showed similar or higher values compared to the controls (Figure 3). GLP1 levels at 30 and 120 min differed between patients with DM1 and the controls (Figure 3C).

In patients with DM1, the 30 min GLP-1 values correlated well with the insulin values (*p* = 0.03). No significant correlations were found for GLP-1 at other times or at any time for GIP. The correlation in control patients with diabetes could not be analyzed because of the small sample size.

GIP did not differ among the NGT, IGT, and DM groups in patients with DM1. GLP1 did not differ between NGT and DM but was significantly lower in patients with IGT than in those with NGT or DM.

### 3.4. Administration of DPP-4 Inhibitor

**Figure 4:**
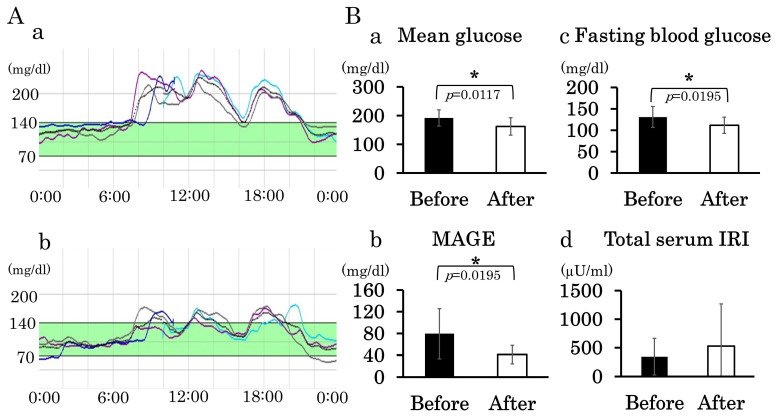
Changes in glucose level variability and glucose metabolism parameters by DPP-4 inhibitors in patients with DM1 complicated with diabetes mellitus. (**A**). Example of CGM profiles before DPP-4 inhibitor administration (**a**) and after DPP-4 inhibitor administration (**b**) in a patient with DM1 complicated with diabetes. (**B**). Shown are (**a**) mean blood glucose, (**b**) MAGE, and (**c**) fasting blood glucose before and after administration, and (**d**) the total amount of insulin. The mean of mean blood glucose, MAGE, and fasting blood glucose is significantly lowered by administering the DPP4 inhibitor. * denotes *p* < 0.05.

CGM was performed before and after the administration of 20 mg teneligliptin in nine patients, whose backgrounds are shown in Supplemental Table S1 (Figure 4A(b)). The mean blood glucose and MAGE levels in all patients after administration were significantly lower than those before administration (Figure 4B(a,b)), and fasting blood glucose levels showed a significant decrease after administration (Figure 4B(c)). The total amount of insulin was not significantly different pre- and post-administration; however, an increase after administration was observed in more than half of the patients (Figure 4B(d)).

Before administration, four patients had low glucose levels of less than 70 mg/dL, and two of them had low glucose levels of less than 60 mg/dL. After administration, three patients had low glucose levels of less than 70 mg/dL, but none had levels of less than 60 mg/dL. There were no significant changes in other blood test results, such as liver enzymes, before and after administration, and no adverse symptoms (including the perception of low glucose levels) were observed.

## 4. Discussion

This is the first study using CGM to analyze the dynamics of daily glucose levels in patients with DM1. The CGM study revealed high glucose sensor values in patients judged as having NGT or IGT by the OGTT. We also found that incretin secretion, the target of DPP-4 inhibitors, was maintained in patients with DM1, and treatment with DPP-4 inhibitors reduced glucose levels and increased insulin secretion in some patients.

Notably, CGM revealed patients with high sensor glucose levels even when the OGTT results indicated NGT or IGT. During CGM, the more times a patient deviates from the reference glucose level, the more advanced the glucose intolerance is [14]. Our result also indicates that even if their HbA1c was below 6.0, there were many patients whose glucose sensor values exceeded 140 mg/dL for a certain period of time (Figure 2 top left). These findings show that even when the OGTT results or HbA1c levels are normal, there may be latent abnormalities in patients’ glucose metabolism. In another cohort, we observed that 8 of 35 non-diabetic DM1 patients (12 normal, 23 borderline) developed diabetes during a 3-year follow-up [8]. Since the post-dinner glucose level was usually the highest, as shown in Figure 1A(b), we believe that CGM may be used for patients with DM1 to detect abnormal glucose metabolism early, using blood glucose levels after dinner as a deciding factor [20].

In the present study, low glucose levels below 70 mg/dL were frequently found in patients with DM1 using CGM (10 out of 29 cases). However, low values in CGM have been reported even in normal subjects up to 2.7% per day below 70 mg/dL [21]. Incidences of severely low glucose levels below 60 mg/dL and 54 mg/dL (cut-off values [21]) were observed in four patients and one patient, respectively. This hypoglycemia may be due to DM1-specific insulin resistance, hyperinsulinemia, and an enhanced insulin response to carbohydrates [1,22,23]. Indeed, the patients with DM1 in the present study showed a higher insulin response during the OGTT than the controls did, which is consistent with previous reports [4,23,24]. However, this finding is contrary to the report [5]. that hypoglycemia is less common in patients with DM1, despite hyperinsulinemia. We believe that this is because CGM was used for long-term continuous observation. In fact, hypoglycemia was observed during the OGTT in only one of the twenty-nine total cases. Frequent episodes of hypoglycemia can precipitate a counterregulatory response during a subsequent hypoglycemic episode [25]. Some patients with DM1 with recurrent unconscious hypoglycemia have also been reported. To capture the characteristics of patients with hypoglycemia, we compared the results of the OGTT, insulin resistance test, insulin secretory capacity, and HbA1c levels, but no apparent differences were observed. Further studies are needed to determine the specific type of DM1 in patients with hypoglycemia and to assess the clinical impact of hypoglycemia.

The present study showed that incretin (GIP and GLP1) secretion was preserved in most patients with DM1. This result justifies the use of inhibitors of DPP-4, an enzyme that breaks down incretin, for DM1 patients. This study confirmed that administering a DPP-4 inhibitor lowered blood glucose levels and increased blood insulin levels. As DPP-4 inhibitors are known to be less likely to cause hypoglycemia [19], our study did not find any exaggeration of low-glucose episodes after the administration.

While DPP-4 inhibitors have shown potential benefits beyond glucose metabolism, including effects on cognitive and cardiac function [26,27], this study was limited in its ability to fully explore these aspects due to the small patient sample and short administration duration. The potential benefits of DPP-4 inhibitors on cognitive and cardiac function are particularly intriguing in DM1 patients, who often experience these impairments. However, DPP-4 inhibitors may also be associated with adverse effects, including skin lesions, immune reactions, and possibly cancer [26,27]. Moreover, it is crucial to evaluate whether DPP-4 inhibitors are more appropriate than other diabetes medications for DM1 patients, taking into account not only their potential benefits but also their cost-effectiveness. Future studies should aim to clarify the role of DPP-4 inhibitors in selected DM1 patients with concomitant diabetes. These studies should involve a larger sample size, extended follow-up periods, and a consistent set of patient characteristics. Comprehensive monitoring of blood glucose and related hormone levels, the use of immunological indicators, and thorough documentation of adverse effects should also be key priorities in future research.

This study has additional limitations. CGM was used for only three days due to the length of hospitalization and the device’s limitations. However, it may be advisable to use CGM measurements at home for an extended period. The use of CGM over an extended period provides more accurate measures of mean glucose levels, time within the normal range, and indicators of hyperglycemia [28,29]. Early detection of risk factors and lifestyle modifications may prevent the conversion of prediabetes to overt diabetes in patients with DM1, as is the case in the general population [30]. Furthermore, we could not elucidate how abnormal blood glucose variability is related to the progression of abnormal glucose metabolism in DM1. Long-term measurements in a larger number of patients are required in the future.

## 5. Conclusions

This is the first report of applying CGM in patients with DM1. Although there are many unanswered questions, this study demonstrates the possibility of using CGM to identify the potential risk of glucose metabolism abnormalities in patients with DM1. In the future, it will be essential to establish how CGM can be used for the early care of diabetes in patients with DM1.

## Figures and Tables

**Table 1:** Background information and glucose and lipid metabolism test results for 29 DM1 patients and 5 disease controls (4 cases of amyotrophic lateral sclerosis, 1 case of multifocal motor neuropathy).

	DM1				Control
	Total	NGT	IGT	DM	
Age (years)	48.9 ± 13.7	54.0 ± 13.3	50.4 ± 13.3	40.7 ± 13.8	65.4 ± 5.8
Sex (men/women)	20/9	4/3	12/3	4/3	3/2
The number of CTG repeat	1228 ± 660	1061 ± 618	1197 ± 455	1500 ± 993	
Body mass index (kg/m^2^)	22.1 ± 4.5	18.2 ± 2.7	23.1 ± 4.5	23.8 ± 3.4	18.8 ± 1.8
HbA1c (%)	5.8 ± 0.8	5.5 ± 0.3	5.5 ± 0.4	6.8 ± 1.0	5.5 ± 0.5
Fasting blood glucose (mg/dL)	94.4 ± 23.8	82.9 ± 8.5	88.2 ± 12.8	119 ± 33.0	113 ± 38.9
Cystatin C (mg/L)	1.04 ± 0.25	1.08 ± 0.33	1.04 ± 0.25	1.01 ± 0.20	0.98 ± 0.21
Low-density lipoprotein cholesterol (mg/dL)	110 ± 35.5	96.7 ± 21.7	116 ± 42.8	113 ± 23.7	84.0 ± 36.4
High-density lipoprotein cholesterol (mg/dL)	45.3 ± 9.7	43.9 ± 7.2	45.6 ± 11.0	46.1 ± 8.5	48.0 ± 19.6
Triglyceride (mg/dL)	166 ± 124	101 ± 27.2	151 ± 113	263 ± 166	216.8 ± 124.5

**Table 2:** Low glucose sensor values with CGM observed in DM1 patients.

	HbA1c (%)	FBS (mg/dL)	120BS (mg/dL)	CGM			
				Lowest (mg/dL)	% < 70 mg/dL	% < 60 mg/dL	
NGT	5.3	77	91	55	9.44	2.62	After breakfast
(6/7)	6.0	100	103	66	2.43	0.00	Midnight
	5.6	82	119	57	1.04	0.52	Before dinner
	5.3	79	92	68	1.04	0.00	Midnight
	5.6	79	91	68	1.04	0.00	After breakfast
	5.6	73	53	65	9.01	0.00	After dinner
IGT	5.1	78	192	68	3.29	0.00	After dinner
(4/15)	5.3	75	175	40	11.09	9.19	Midnight
	5.7	82	163	59	8.15	0.52	Midnight
	5.8	89	129	62	2.95	0.00	After dinner

120BS: Blood sugar value at 120 min with OGTT. % < 70 mg/dL and % < 60 mg/dL: percentage of glucose sensor values below 70 mg/dL and 60 mg/dL, respectively.

## Data Availability

The data that support the findings of this study are available on request from the corresponding author, M.P.T., upon reasonable request.

## Supplementary Materials

The following supporting information can be downloaded at: https://www.mdpi.com/article/10.3390/jcm13175252/s1, Table S1. Background information on DM1 patients with diabetes mellitus treated with DPP-4 inhibitors.

## Abbreviations

ALS, amyotrophic lateral sclerosis; CGM, continuous glucose monitoring; DM1, myotonic dystrophy type 1; DPP-4, dipeptidyl peptidase 4; GLP-1, glucagon-like peptide-1; GIP, gastric inhibitory polypeptide; IBW, ideal body weight; IRI, immunoreactive insulin; MAGE, mean amplitude of glucose value; OGTT, oral glucose tolerance test; SMBG, self-monitoring of blood glucose.
