# Supplementary material for: Investigation of Glucose Metabolism by Continuous Glucose Monitoring and Validation of Dipeptidyl Peptidase 4 Inhibitor Use in Patients with Myotonic Dystrophy Type 1
Source: J Clin Med. 2024 Sep 5;13(17):5252. doi: 10.3390/jcm13175252 (PMC11396113; doi:10.3390/jcm13175252)
Supplement: Supplementary file 1 [file jcm-13-05252-s001.zip › jcm-3157036-supplementary.pdf]

Supplemental Table S1. Background information on DM1 patients with diabetes mellitus treated with DPP-4 inhibitors

Characters	DM (n=9)
Age (years)	46.6±7.9
The number of CTG repeat	1625±785
HbA1c (%)	7.4±0.5
Fasting blood glucose (mg/dl)	131±24.5
75gOGTT 120min blood glucose (mg/dl)	283±63.6
